# Enhanced multi-class object detector for bone fracture diagnosis with prescription recommendation

**DOI:** 10.3389/frai.2025.1692894

**Published:** 2026-01-12

**Authors:** Daudi Mashauri Migayo, Shubi Kaijage, Stephen Swetala, Devotha G. Nyambo

**Affiliations:** 1 School of Computational and Communication Science and Engineering, The Nelson Mandela African Institution of Science and Technology, Arusha, Tanzania; 2 Department of Business Administration, Tanzania Institute of Accountancy (TIA), Dar es Salaam, Tanzania; 3 Department of Orthopedic and Trauma Surgery, Bugando Medical Centre, Mwanza, Tanzania

**Keywords:** bone fracture, object detection, adaptive anchoring, prescription, Tanzania

## Abstract

Bone fractures are among the most prominent injuries in the modern world that affect all ages and races. Traditional treatment involves radiographic imaging that relies heavily on radiologists manually analyzing images. There have been efforts to develop computer-aided diagnosis tools that employ artificial intelligence and deep learning approaches. Existing literature focuses on developing tools that only detect and classify bone fractures, rather than addressing the broader issue of bone fracture management. However, evidence of scholarly works that include treatment recommendations is still lacking. Furthermore, deep learning-based object detectors that achieve state-of-the-art results are computationally expensive and considered as black-box solutions. Developing countries, such as Sub-Saharan Africa, face a shortage of radiologists and orthopedists. For this reason, this paper proposes a methodological approach that uses a more efficient object detection model to diagnose long bone fractures and provide prescription recommendations. An enhanced anchoring process, known as adaptive anchoring, is proposed to improve the performance of the Regional Proposal Network and the object detection model. A Faster R-CNN model with ResNet-50/101 and ResNext-50/101 backbones was used to develop an object detection model that uses X-ray images as input. To understand and interpret the model’s decision, a Gradient-based Class Activation Mapping method was used to assess the model’s learnability. The results indicate that the proposed adaptive anchoring approach can improve computational efficiency, reducing training time by up to 29% compared to the traditional approach. Model accuracy during training and validation ranged between 94% and 98%. Overall, adaptive anchoring performed better when applied with the ResNet-101 backbone, yielding an Average Precision of 92.73%, an F1 score of 96.01%, a precision of 96.80%, and a recall of 95.23%. The study provides valuable insights into the use of computationally efficient deep learning models for medical recommendation systems. Future studies should develop models to diagnose fractures using input images from various modalities and to provide prescription recommendations.

## 1 Introduction

**Figure 1:**
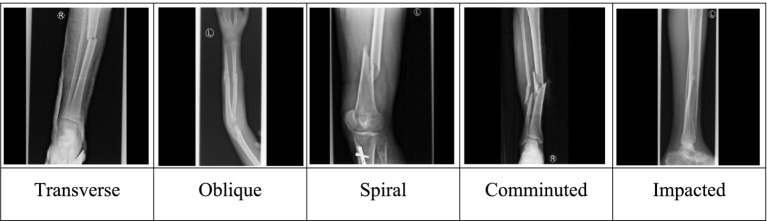
Common fracture patterns.

Bones constitute part of the skeletal system, protect internal organs, and facilitate movements in vertebrate animals. However, human bones are prone to fractures from automobile accidents and falls. The World Health Organization (WHO) estimates the loss of 1.19 million lives, between 20 and 50 million non-fatal injuries, costing 3% of gross domestic product yearly, due to road traffic crashes (WHO, 2023). Common fracture patterns that medical professionals are likely to encounter in their daily work include transverse, oblique, spiral, comminuted, greenstick, and impacted fractures, as shown in Figure 1. Fibula/tibia (leg) and femur (thigh) fractures are the most common fractures in Africa, classified by fracture location (Pouramin et al., 2019).

While traditional fracture treatment relies heavily on radiographic imaging, this approach has limitations. Despite its remarkable capabilities, the human eye often struggles to detect minor fractures (Yadav and Rathor, 2020). Furthermore, doctors who frequently deal with emergencies can be hindered by fatigue (Tanzi et al., 2020). These limitations underscore the pressing need for more advanced tools, such as computer-aided diagnosis (CAD), in the treatment of fractures. Applying CAD tools powered by deep learning models has significantly enhanced the performance of radiographic diagnosis (Lindsey et al., 2018). Applying deep learning approaches has yielded state-of-the-art performance results in fracture diagnosis (Ma and Luo, 2021). These advancements hold immense promise for the future of healthcare. The success of deep learning in diagnosis led to the introduction of recommendation systems to enhance personalized healthcare (Lichtner et al., 2023; Nayak et al., 2023; Wang and Qian, 2021). Developing countries, such as those in SSA, face a shortage of radiologists (Laage Gaupp et al., 2019) and orthopedists (Wilhelm et al., 2017). Applying deep learning models to fracture diagnosis—including prescribing recommendations—may significantly enhance healthcare delivery in resource-limited environments. However, deep learning models that guarantee state-of-the-art performance results are known to be computationally expensive (Thompson et al., 2023). There have been efforts to make deep learning models smaller, faster, and much better than traditional ones (Menghani, 2023). Furthermore, deep learning models are known to lack transparency and explainability in their predictions. This has become a significant concern for practitioners when they cannot tell how models make predictions and the key features that lead to a specific decision.

This paper proposes an enhanced multi-class object detection model with adaptive anchoring for fracture diagnosis, with prescription recommendations as a second opinion to radiologists and surgeons. Radiologists labeled the collected X-ray images, and orthopedists suggested prescription recommendations. The Regional Proposal Network (RPN) was modified to guide the anchoring process and avoid searching areas where fractures are unlikely to be located. This study selects the standard surgical methods based on three assumptions to implement recommendations. First, patients are skeletally mature, and X-ray images of only adult patients are included. Second, the distal neurovascular status is intact, allowing for limb salvage. Third, fractures are classified as open or closed, from Gustilo-Anderson I to IIIA. Impacted fractures are typically treated with immobilization, such as casting or splinting. Other standard surgical methods are intramedullary nailing (Shen and Tejwani, 2024) and plate osteosynthesis (Hansmann, 1886). Fracture patterns and surgeon preferences are often applied to select the optimal treatment of bone fractures (Hurley et al., 2023). To address explainable artificial intelligence (XAI), a Gradient-based Class Activation Mapping (Grad-CAM) method was used to examine how the model makes predictions from input images. The main contributions of this paper can be summarized in four aspects:

- The demographic of bone fractures to characterize the distribution in developing countries is documented.
- A modified anchoring process, called adaptive anchoring, to improve the RPN and performance of the object detection model is proposed.
- An enhanced multi-class object detector using bounding box regression is trained for fracture diagnosis with prescription recommendations.
- The Grad-CAM method is applied to explain how the model makes predictions from the given input images.

The remaining part of the paper is organized as follows: Section 2 presents the materials and methods used in this study. Section 3 presents the results of this study’s discussions. Section 4 provides a conclusion and recommends future research.

## 2 Materials and methods

### 2.1 Ethics statement

This study was approved by the ethics committee governed by three institutions: The Centre for Education Development in Health (CEDA), Kibong’oto Infectious Diseases Hospital (KIDH), and the Nelson Mandela African Institution of Science and Technology (NM-AIST), letter No: KNCHREC/00068/11/2022 issued January 18th, 2023. Multi-view X-ray images were collected from the Kilimanjaro Christian Medical Centre (KCMC) in Kilimanjaro and the Muhimbili Orthopedic Institute (MOI) in Dar es Salaam, Tanzania.

### 2.2 Data collection

Digital Imaging and Communication in Medicine (DICOM) format was used to store captured X-ray images. Images were stored together with the patient’s medical records in the health information system. An Open Health Imaging Foundation (OHIF) web platform was used to extract and convert DICOM images. The photos were saved in JPEG and PNG formats, with randomly generated file names for de-identification. A separate index file was created to map images and their corresponding labels. Bone fracture labeling was conducted on long bones, including the radius, ulna, femur, and tibia, to annotate the presence and anatomical locations of fractures. Five board-certified senior radiologists independently reviewed images for fracture classification. The standard radiological criteria for fracture diagnosis, including assessment of cortical disruption and displacement, were applied during labeling. To assess inter-rater reliability, Cohen’s Kappa coefficient was calculated and found to be 0.85, indicating strong agreement. An orthopedic surgeon lastly reviewed the images and included the treatment recommendations.

### 2.3 Dataset

**Figure 2:**
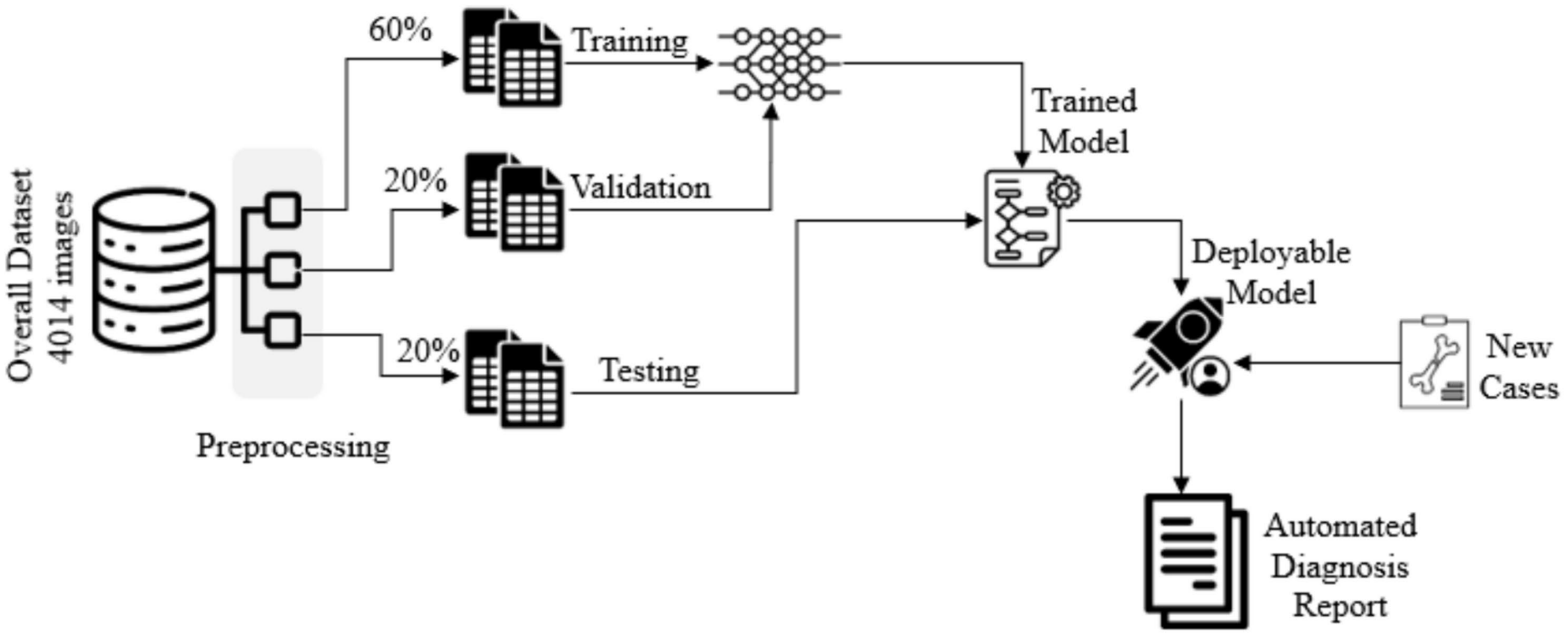
Training pipeline of the multi-class object detector.

The Robo flow online tool was used to draw bounding boxes on X-ray images and generate Tensor Flow Object Detection format files to train an object detection model. A total of 4,014 images of long bones, comprising 864 forearms (ulna and radius), 414 upper arms (humerus), 1,530 legs (fibula and tibia), and 1,206 thighs (femur), were collected between October 2022 and September 2023. The dataset was split into three non-overlapping image sets with a ratio of 60:20:20 for training, validation, and testing, as recommended for studies involving deep learning models (Muraina, 2021). Stratified 10-fold cross-validation was used to address class imbalance and ensure robust results. Figure 2 summarizes the training pipeline of an object detector.

Data augmentation techniques were applied during preprocessing to improve model generalization. Variations of the same image were created through geometric transformations and colour transformations. Geometric transformations include rotation, random cropping (80%), scaling, and horizontal flipping (*p* = 0.5). Colour transformations include brightness, contrast, and saturation adjustments within ±20%. These data augmentation techniques simulate real-world variations, thereby enhancing the model’s robustness. Augmentation was confined to the training split in each fold, with no leakage across folds. Table 1 summarizes the dataset and augmentation ranges for each class, grouped according to the corresponding bone fracture treatment.

**Table 1:** Dataset distribution and augmentation range for each class.

Class	Recommendations	Total	Testing	Training	Augmented
I	ORIF FAD	522	104	314	1,620
II	ORIF IMN	1,004	201	602	3,020
II	ORIF plate	1,485	297	891	4,400
IV	Casting	1,004	201	602	4,000

The classes pose a severe imbalance challenge, especially given that class I accounts for around 13% of the total dataset. Per-class support and cost-sensitive strategy were used to rebalance the outcomes of model decisions. Underrepresented classes were penalised more heavily than overrepresented classes. Table 2 summarizes the class support and weights used during sampling to handle class imbalances.

**Table 2:** Class support and class weight to address the imbalance challenge.

Class	Recommendations	Class support	Class weight
I	ORIF FAD	314	1.76
II	ORIF IMN	602	0.92
II	ORIF plate	891	0.62
IV	Casting	602	0.92

### 2.4 Treatment recommendations

Throughout the study, standard surgical methods were applied to implement the recommendations. However, in some cases, fracture management may vary depending on resource availability and the surgeon’s preference. Table 3 summarizes standard surgical methods applied to implement treatment recommendations for bone fractures.

**Table 3:** Surgical methods for treatment recommendations.

Fracture location			
Skeletal part	Proximal third	Mid-third	Distal third
Femur (thigh)	ORIF FAD	ORIF IMN	ORIF plate
Fibula and Tibia (leg)	ORIF plate	ORIF IMN	ORIF plate
Humerus (upper arm)	ORIF plate	ORIF IMN	ORIF plate
Urna and radius (forearm)	ORIF plate	ORIF plate	ORIF plate
Any impacted fracture	Casting	Casting	Casting

We implemented a hierarchical rule-based classifier to map fractures into four treatment-strategy categories (casting, ORIF-FAD, ORIF-IMN, and ORIF-Plate). The model uses structured descriptors derived from imaging annotations—including fracture location, pattern complexity, displacement, comminution, and morphological stability tags—to evaluate eligibility for each treatment class. Each class is associated with an inclusion–exclusion rule set derived from established orthopedic taxonomies. These rules do not produce clinical recommendations but serve as deterministic criteria for benchmarking automated labeling and evaluating model consistency relative to expert-assigned categories.

### 2.5 Model selection

An object detection model for fracture diagnosis was implemented using a deep convolutional neural network as the backbone network. ResNet (He et al., 2016) was implemented as the backbone, as it is among the prominent models for fracture detection (Meena and Roy, 2022). An object detector containing the Faster R-CNN model with a ResNet backbone for feature extraction guarantees a better performance (Tahir et al., 2021).

### 2.6 Adaptive anchoring

This paper proposes an adaptive anchoring Faster R-CNN for bone fracture diagnosis. After scrutinizing X-ray images containing long bone fractures, it is revealed that the Region of Interest (ROI) is often positioned relatively close to the center of the image. The number of anchors is significantly reduced by focusing on a small area. Therefore, the overall efficiency of the anchoring process can be improved.

**Figure 3:**
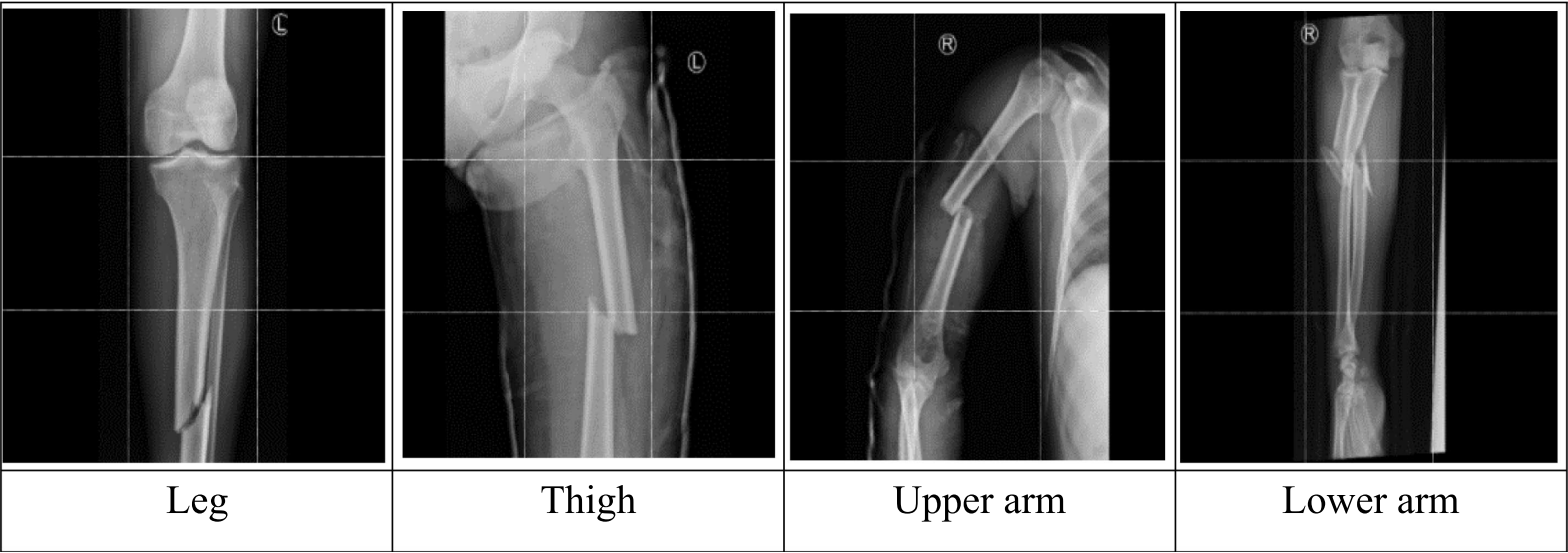
Location of fractured regions.

Input images are loaded, and features are extracted using a backbone network. The image features output from the backbone network are considered inputs for the RPN network. Conventionally, RPN scans the input image and generates anchors across the image. This paper introduces adaptive anchoring to guide the anchoring process and avoid areas where fractures are unlikely to occur. Given the nature of X-ray images containing long-bone fractures, the fractured regions can be located within an area after omitting a portion on either side of the image, as well as at the top and bottom. Figure 3 illustrates the possible location of fractured regions after dividing an image into nine sectors.

Given an image centered at (x,y) with height h and width w , by avoiding 33% of the width on either side and 16.5% on the top and bottom, the area for RPN to scan can be guided within the four coordinates beginning with the top-left corner by considering the following Equations 1–4.

A=(x−0.17w,y+0.34h) (1)

B=(x+0.17w,y+0.34h) (2)

C=(x+0.17w,y−0.34h) (3)

D=(x−0.17w,y−0.34h) (4)

**Figure 4:**
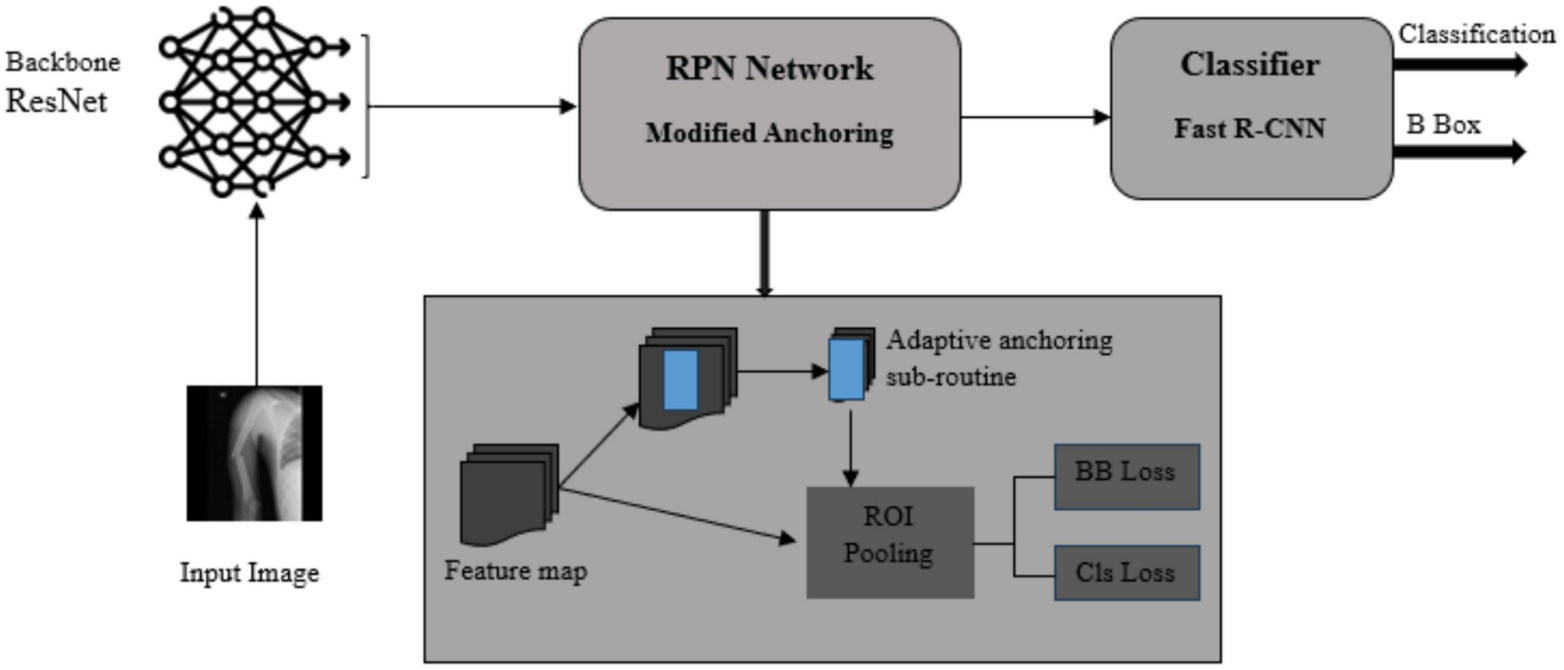
Functional structure.

Algorithm 1 summarizes the entire adaptive anchoring process. The input images are in grayscale and have low brightness, which hinders feature extraction. The signal-to-noise ratio and detection features can be improved by applying brightness normalization to the images. This approach has been used in similar studies that apply object detection for bone fracture diagnosis (Wang and Huang, 2022). Figure 4 provides the functional structure of the proposed object detection model.

**ALGORITHM 1:**
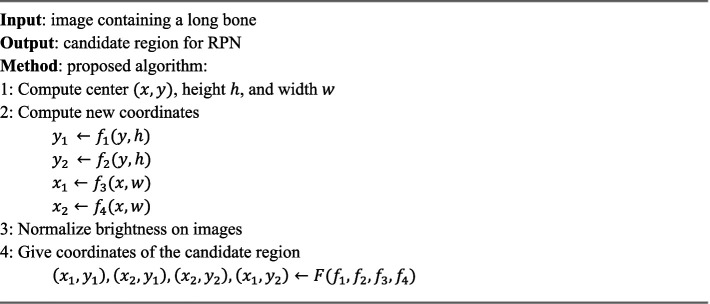
Adaptive anchoring to guide RPN.

Equation 5 was used to implement brightness normalization. The scaling factor is given as K , where r represents the value of a pixel in a particular image, $r_{\min}$ is the minimum pixel value, and $r_{\max}$ is the maximum pixel value. Pixel values are normalized within the range of [0, 1] with a scaling factor K applied to enhance brightness (setting K > 1) or reduce brightness (setting K < 1), otherwise set to 1 in deal cases.

$$S=K*\frac{r-r_{\min}}{r_{\max}-r_{\min}}$$ (5)

### 2.7 Evaluation metrics

The Average Precision (AP) and F1 score were used as evaluation criteria in this study, along with accuracy, precision, and recall. These metrics are commonly used in similar studies involving object detection models.

### 2.8 Implementation details

Python was used to code the experiments in this study, utilizing Jupyter notebooks within a TensorFlow framework. Training and testing of the object detection models were conducted on a personal computer. The PC features a 1 TB hard disk, 16GB of RAM, and an Intel Core i7 processor. NVIDIA GeForce GTX 1650 Ti GPU-accelerated graphics with Max-Q design. The operating system was a 64-bit version of Windows 11 Pro, version 23H2.

The classification and bounding-box regression losses were combined to form a multi-task loss function. The model’s training parameters were updated using an SGD optimizer configured with a weight decay of 0.0001 and a momentum of 0.9. The learning rate was set to 0.001 and scheduled with weight decay, which reduces the rate by 0.1 after every 10 iterations.

### 2.9 Explainable artificial intelligence

Grad-CAM was integrated into the object detector to visualize how regions of the input image contributed to the predictions made. Initially, the model performed a forward pass, and the last convolutional layer produced feature maps and generated predictions. Then, the gradient of the class score was computed with respect to feature maps. Afterward, the global average of the gradients was used to calculate the weight of each channel in the feature map by using Equation 6.

$$\propto_{c}^{k}=\frac{1}{Z}\sum_{i,j}\frac{\partial y_{c}}{\partial A_{i,j}^{k}}$$ (6)

Where $y_{c}$ represents the score for the class C , $A_{i,j}^{k}$ signifies the activation at the location (i,j) for channel k , and Z is the normalization factor. Computed weights were used to combine weighted feature maps and generate a Grad-CAM heatmap using Equation 7.

$$\text{Grad}\_\mathrm{CAM}=\text{ReLU}(\sum_{k}\propto_{c}^{k}A^{k})$$ (7)

Generated heatmaps were resized to match the input images’ sizes, and each map was overlaid on the original image to produce the visual representation. The visual representation shows which regions of the input image the model focused on during fracture detection.

## 3 Results and discussions

### 3.1 Comparative analysis of recent literature

The literature shows efforts to improve fracture diagnosis using AI and ML tools. Initially, classical machine-learning approaches were applied to detect and classify fractures (Johari and Singh, 2018; Myint et al., 2018). Later, deep learning achieved cutting-edge results and was expected to surpass human capabilities in radiographic imaging (Ma and Luo, 2021). Afterward, researchers sought to improve the efficiency and performance of fracture-diagnosis models.

Different techniques have been applied to improve efficiency, for instance, an anchor-based model (Qi et al., 2020), a crack-sensitive model (Ma and Luo, 2021), a feature ambiguity model (Wu et al., 2021), automated preprocessing (Wang and Huang, 2022), and a two-stage model (Yang et al., 2022). Other techniques focus on improving performance and efficiency, such as ensemble-based neural networks (Ghosh et al., 2021), and a guided anchoring model (Xue et al., 2021). Researchers applied pre-trained deep learning models, fitted to smaller datasets, to identify fractured radiographs from non-fractured ones (Nikhil et al., 2023; Kandel et al., 2020). This paper contributes to the existing literature by proposing an alternative approach that aims to improve the efficiency and performance of models. By modifying the conventional anchoring process and adapting it to the task at hand, detection models can be made more efficient. Avoid searching for objects that are never located, which saves computational power and improves overall performance. Furthermore, the paper provides fracture demographics classified by age, fracture location, and mechanism of injury. This broadens understanding of the problem and offers valuable insights into targeted measures to either eliminate or reduce it in developing countries. Table 4 summarizes the main contribution of this paper relative to existing literature.

**Table 4:** Comparative assessment of existing literature and main contributions.

References	Approach	Main contribution
Qi et al. (2020)	Anchor-based model	Improved performance
Ghosh et al. (2021)	Ensemble neural networks	Improved performance, efficiency
Xue et al. (2021)	Guided anchoring	Improved performance, efficiency
Ma and Luo (2021)	Two-stage crack-sensitive	Improved performance
Wu et al. (2021)	Feature ambiguity preprocessor	Improved performance
Wang and Huang (2022)	Attention mechanism	Improved performance
Yang et al. (2022)	Cascade models	Improved performance
Nikhil et al. (2023); Kandel et al. (2020)	Transfer learning	Improved performance, efficiency
Proposed approach	Multi-class model with adaptive anchoring	Improved performance, efficiency, prescription recommendations, and bone fracture demographics.

### 3.2 Bone fracture trends and distribution

Table 5 presents an overview of fracture distribution disaggregated by gender and age. Out of 1,410 patients, 63% were males, with the majority of fractures occurring between the ages of 25 and 54. The fewest cases were observed among female patients aged 18 to 24.

**Table 5:** Fracture distribution disaggregated by gender.

Fracture demographics		Age (in years)						Total
		18–24	25–34	34–44	45–54	55–64	≥ 65	
Gender	Female	63	78	99	104	94	84	522
	Male	98	266	213	133	98	80	888
Total		161	344	312	237	192	164	1,410

Table 6 presents the distribution of fractures by fracture location. Leg-dominated long bone fractures account for 36.2% of the entire distribution. Most fractures occurred between the ages of 25 and 44, accounting for 52.3% of leg fractures, indicating that youths are more affected by leg fractures, while elders are more affected by femur fractures.

**Table 6:** Fracture site distribution categorized by age.

Fracture location		Age (in years)						Total
		18–24	25–34	34–44	45–54	55–64	≥ 65	
Femur	Female	21	20	23	29	30	33	156
	Male	30	67	49	36	32	32	246
	Total	51	87	72	65	62	65	405
Leg	Female	15	32	40	42	26	20	175
	Male	39	109	86	54	28	19	335
	Total	54	141	126	96	54	39	510
Forearm	Female	13	16	19	18	22	19	107
	Male	21	54	41	23	24	18	181
	Total	34	70	60	41	46	37	288
Upper arm	Female	14	7	10	11	11	9	62
	Male	8	22	20	14	12	8	84
	Total	22	29	30	25	23	17	146
Multiple	Female	0	3	7	4	5	3	22
	Male	0	14	17	6	2	3	42
	Total	0	17	24	10	7	6	64
Total		161	344	312	237	192	164	1,410

The mechanism of injury, as presented in Table 7, is primarily RTI, which accounts for 48.7% of the total cases, with the majority of these cases reported among individuals aged 25–54. This indicates that the working-age population suffers the most from RTIs. On the other hand, falls are the leading cause of injury among older people, and the risk increases with age. The youth suffer the least from falls, with the fewest cases among those aged 18 to 24. The following section presents the empirical results of performance evaluations of the proposed multi-class object detection model.

**Table 7:** Mechanism of injury disaggregated by age.

Mechanism of injury		Age (in years)						Total
		18–24	25–34	34–44	45–54	55–64	≥ 65	
Bad fall	Female	25	25	32	43	43	43	211
	Male	39	74	62	54	44	41	314
	Total	64	99	100	97	87	84	531
RTI	Female	28	48	63	51	31	20	241
	Male	43	162	125	65	32	19	446
	Total	71	210	188	116	63	39	687
Others^*^	Female	10	5	4	10	20	21	70
	Male	16	30	20	14	22	20	122
	Total	26	35	24	24	42	41	192
Total		161	344	312	237	192	164	1,410

* Including animal attacks, bicycle, lifting, intentional, or other.

This study discovered that male patients are significantly more affected by fractures than their female counterparts. A possible explanation is that in developing countries like Sub-Saharan Africa, males are more likely to engage in high-risk outdoor activities. Most fractures in males occur in patients between the ages of 25 and 54 and decrease significantly with age. Fractures in female patients do not vary considerably with age but do increase slightly with age. One interesting finding is that the distribution of fractures is comparable between male and female patients in old age. Several factors could explain this observation. First, high-risk outdoor activities in males tend to decrease with age. Second, in old age, falls are the primary contributor to injuries, affecting both males and females equally. Third, both males and females are affected by osteoporosis, which increases skeletal fragility and the risk of fractures.

Other studies have similarly identified the dominance of males in fracture patients (Chen et al., 2024). However, in some regions with different socioeconomic conditions, the number of female patients is significantly higher than that of males (Bergh et al., 2021). This discrepancy could be attributed to regional socioeconomic status, which determines the nature and type of functions performed by males and females in their communities. Results from this study indicate that the leading cause of long bone fractures that affect the working-age population is road traffic injuries. The working-age group tends to suffer most from RTI because of involvement in high-risk outdoor activities. RTI poses a severe economic threat in lower socioeconomic countries like those in Sub-Saharan Africa, affecting individuals, families, and nations at large. The WHO estimates a 3% loss of gross domestic product in most countries due to road traffic crashes (WHO, 2023).

### 3.3 Training and inference time of enhanced object detector

The effectiveness of the proposed adaptive anchoring approach was evaluated for each image by comparing the training and prediction times. Results were benchmarked against the standard Faster R-CNN model with ResNet-50/101 and ResNext-50/101 backbone networks. The Faster R-CNN training time ranged from 50 to 70 h, and the inference time per test image was 110–196 milliseconds on the GPU and 280–590 milliseconds on the CPU. The proposed Faster R-CNN with adaptive anchoring achieved training times of 40–50 h, and inference time per test image ranged from 105 to 192 milliseconds on the GPU and 283 to 496 milliseconds on the CPU. This result implies that the proposed approach can improve training time by up to 29%. Table 8 summarizes the training and inference times of the models using four backbones.

**Table 8:** Training time and inference time of object detection models.

Network	Backbone	Training time	Inference time (GPU, CPU)
Faster R-CNN	ResNet-50	52 Hrs	(110, 280) ms
	ResNet-101	63 Hrs	(170, 490) ms
	ResNext-50	61 Hrs	(125, 305) ms
	ResNext-101	69 Hrs	(196, 504) ms
Faster R-CNN with adaptive anchoring	ResNet-50	40 Hrs	(105, 283) ms
	ResNet-101	45 Hrs	(164, 488) ms
	ResNext-50	42 Hrs	(127, 297) ms
	ResNext-101	50 Hrs	(192, 496) ms

### 3.4 Performance evaluation

The proposed approach was applied to train a model, achieving an accuracy of 94% to 98%. The model’s learning ability was notably good as the loss index converged with increasing training iterations. The loss index plateaued at 0.25. The box regression loss was 0.12, and the class accuracy was 0.96. The results of the proposed adaptive anchoring approach were benchmarked with a standard Faster R-CNN model. Table 9 presents the average performance results of the proposed approach after fine-tuning the detection models across the 10 data splits. Overall, better performance was observed when adaptive anchoring was applied with a ResNet-101 backbone, yielding an AP of 92.73%, an F-1 score of 96.01%, a precision of 96.80%, and a recall of 95.23%.

**Table 9:** Average performance results of object detection models.

Network	Backbone	Precision	Recall	F-1	AP
Faster R-CNN	ResNet-50	0.8713	0.8550	0.8631	0.8656
	ResNet-101	0.8888	0.8912	0.8900	0.8499
	ResNext-50	0.8823	0.8348	0.8579	0.8774
	ResNext-101	0.9020	0.8591	0.8800	0.8433
Faster R-CNN with adaptive anchoring	ResNet-50	0.9599	0.9356	0.9476	0.8997
	ResNet-101	0.9680	0.9523	0.9601	0.9273
	ResNext-50	0.9482	0.9038	0.9255	0.8814
	ResNext-101	0.9713	0.9330	0.9517	0.8984

The results in this sub-section suggest that the RPN in object detection models can be adapted to improve performance on a specific task. Training time and overall performance significantly improve by avoiding searching areas where objects are never in the images. The performance was further assessed stratified by site where images were acquired. AP and F-1 scores are reported as means and bootstrapped with 95% confidence intervals (CIs). Results indicate minor variations in F-1, ranging from 0.2% to 0.8%, with data from Muhimbili National Hospital on the positive side. The AP ranged from 0.2% to 0.9% across data from the two cohorts. The results indicate that the proposed model can be deployed in regional hospitals while maintaining the desired output level. Table 10 presents performance results stratified by the site of acquisition.

**Table 10:** Average performance stratified by acquisition site.

Network	Backbone	Muhimbili		KCMC	
		F-1 (95% CI)	AP	F-1	AP
Faster R-CNN	ResNet-50	85.7 (81.4–90.1)	85.6 (79.8–91.2)	85.2 (80.8–89.4)	85.3 (78.9–90.7)
	ResNet-101	88.7 (83.6–91.4)	85.1 (78.7–90.6)	87.9 (82.6–90.8)	84.9 (82.9–89.8)
	ResNext-50	86.1 (83.4–90.7)	88.4 (83.9–92.3)	86.2 (82.9–91.0)	89.3 (83.1–91.7)
	ResNext-101	87.9 (82.6–91.7)	84.8 (77.6–89.8)	86.7 (81.9–90.8)	84.2 (78.1–90.2)
Faster R-CNN with adaptive anchoring	ResNet-50	95.1 (90.3–97.8)	89.2 (85.6–93.4)	94.6 (89.9–96.8)	89.9 (84.7–94.1)
	ResNet-101	96.4 (91.5–98.9)	93.1 (88.9–98.4)	96.1 (91.2–99.1)	92.8 (88.6–97.8)
	ResNext-50	91.8 (88.2–95.7)	88.2 (83.5–92.5)	91.6 (87.9–94.6)	87.9 (82.8–91.7)
	ResNext-101	95.6 (91.2–96.8)	89.7 (86.4–94.1)	94.9 (90.6–97.2)	89.2 (84.7–93.8)

Afterward, a Faster R-CNN model with a ResNet-101 backbone was applied to assess the model’s ability in recommending prescriptions. Unseen data were organized into four classes corresponding to each prescription recommendation. On average, precision was 86.37%, recall was 84.86%, and average precision was 85.19%. These performance results are desirable for a well-trained object detection model applied to detect bone fractures. Table 11 summarizes the model’s average performance in prescribing recommendations.

**Table 11:** Average performance results of the model’s ability to recommend prescriptions.

Class	Recommendations	Precision	Recall	F-1	AP
I	ORIF FAD	0.7492	0.7168	0.7326	0.7264
II	ORIF IMN	0.8946	0.8892	0.8918	0.8904
II	ORIF Plate	0.9224	0.9088	0.9155	0.9044
IV	Casting	0.8889	0.8799	0.8843	0.8864
	Average	0.8637	0.8486	0.8561	0.8519

As shown in the table, the performance of Class I recommendations was not particularly impressive, with an average precision of 72.64%. This can be explained by the limited number of data instances resulting in ORIF FAD, where cases accounted for approximately 13% of the total. Class II accounted for 25%, Class III for 37%, and Class IV for 25% of the total. This implies that the more training data and representative data are available, the better the overall model’s performance.

**Figure 5:**
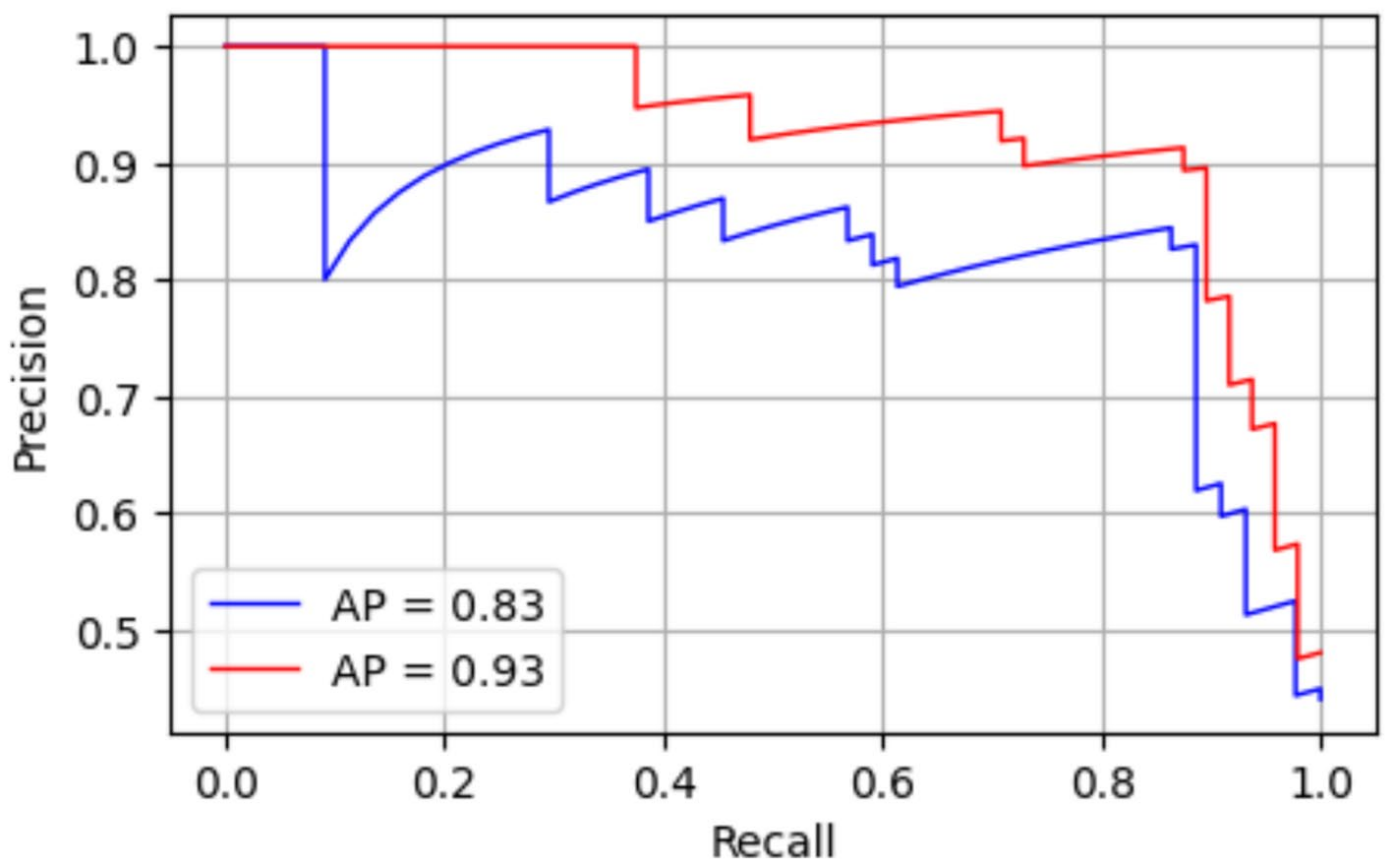
PR curve for standard R-CNN (blue) and adaptive (red).

**Figure 6:**
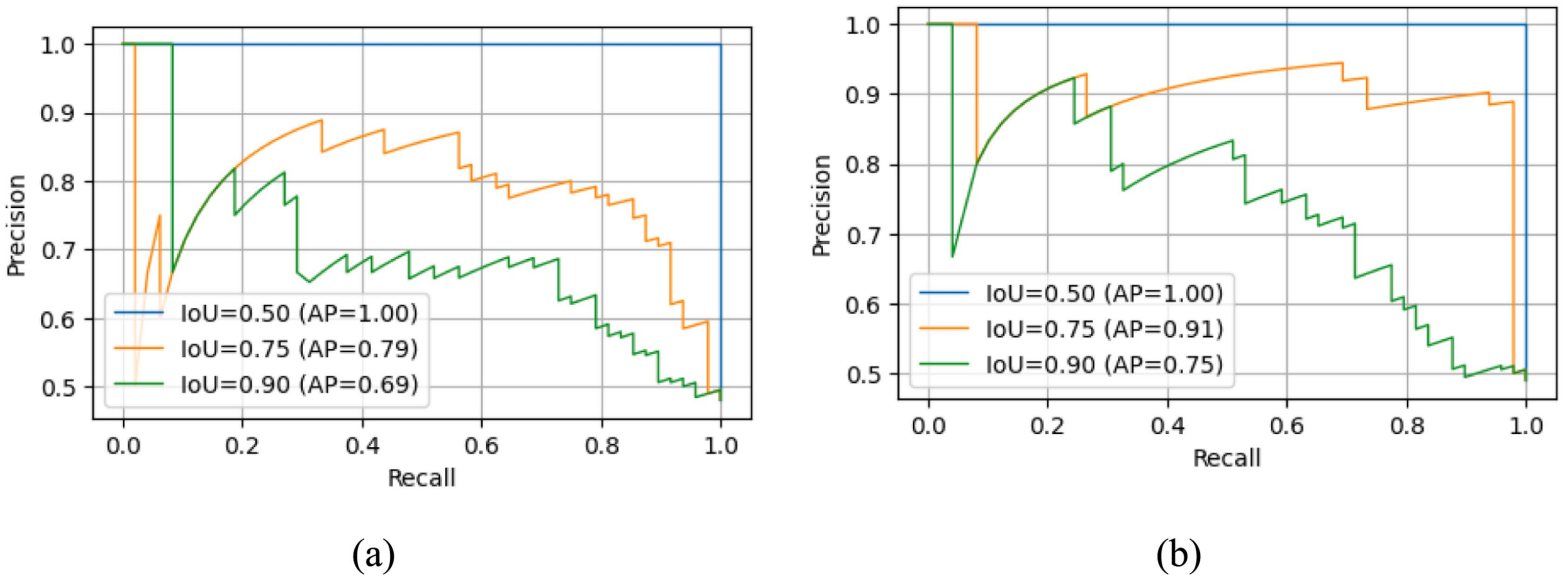
AP at multiple IoU thresholds for standard Faster R-CNN **(a)** and adaptive anchoring of Faster R-CNN **(b)**.

The Precision Recall (PR) for both standard Faster R-CNN and Faster R-CNN with adaptive anchoring is given in Figure 5. The proposed adaptive anchoring approach attained an area under the curve (AUC) of 93%, surpassing the standard R-CNN by 10%. Figure 6 shows the AP for both standard and adaptive anchoring of Faster R-CNN at multiple intersection-over-union (IoU) thresholds. Both models achieved comparable performance on the test set when the threshold was lenient. When the threshold is strict (@0.75), the standard R-CNN achieved an AUC of 79%, which is lower than the 91% achieved with adaptive anchoring. With a more stringent threshold (@0.90), the standard R-CNN attained an AUC of 69%. This implies that the proposed adaptive anchoring approach outperforms standard anchoring, even as thresholds become increasingly stringent.

The proposed adaptive anchoring method reduces the total number of anchors per input image from 160,000 to 53,000 (67% reduction), resulting in a 29% reduction in training time. These results indicate that standard R-CNN with conventional anchor configurations is redundant. The adaptive anchoring approach achieves high performance with significantly fewer anchors, thereby reducing computational cost. Table 12 summarizes anchor density for each FPN layer for both standard Faster R-CNN and adaptive anchoring Faster R-CNN.

**Table 12:** A summary of anchor density reduction.

FPN level	Standard anchoring	Adaptive anchoring	Reduction
P2	40,000	13,200	−40%
P3	10,000	3,300	−67%
P4	2,500	850	−66%
P5	625	200	−68%
P6	169	52	−62%
Positions	53,294	17,602	−70%
Anchor density	159,882	52,806	−67%

### 3.5 Deployment and latency

The developed model was embedded in a web-based system prototype and deployed on a server with an Intel Core i7-8565U (8 cores @ 1.99 GHz), 128 MB UHD Graphics, 8 GB RAM, and a 477 GB SSD, running Windows 11 Pro v23H2. The connection between the client node and the server node occurred over a 100 Mbps LAN. Measurements of latency reflect single-user end-to-end latency unless otherwise stated. Table 13 summarizes the end-to-end web request latencies between a client and server. Results include aggregated latency values and distribution percentiles, including preprocessing from the picture archiving and communication system (PACS) to the model decision.

**Table 13:** End-to-end latency from PACS to decision.

Metric	Value (in seconds)
Average latency	203
Median (P50)	207
P90	277
P95	293
P99	299

The minimum hardware requirements were determined by progressively reducing resources until the system exceeded the expected latency threshold of 420 s. A quad-core Intel i5 laptop CPU with 8 GB of RAM is sufficient to host the prototype, achieving a median latency of 300 s. Table 14 summarizes the minimum hardware requirements.

**Table 14:** Minimum hardware requirements for acceptable performance.

Resource tier	CPU	RAM	Storage	Achieved latency
Recommended	4 cores @ ≥ 2.0 GHz	8 GB	SSD	300 s
Minimum	2 cores @ ≥ 1.8 GHz	4 GB	SSD	390 s
Below minimum	2 cores @ < 1 GHz	2GB	HDD	> 600 s

The proposed system achieves a reasonably low end-to-end latency (median: 207 s, P95: 293 s) when deployed on an 8-core Intel server with 8 GB of RAM. Even under reduced configurations (quad-core CPU, 4 GB RAM), the prototype remains functional, with a median latency of 300 s, making it ideal for practical deployment on low-cost hardware. However, the full implementation of the proposed system prototype is sought to be a module accessed through the existing EMR system in local hospitals.

### 3.6 Interpreting model decisions

**Figure 7:**
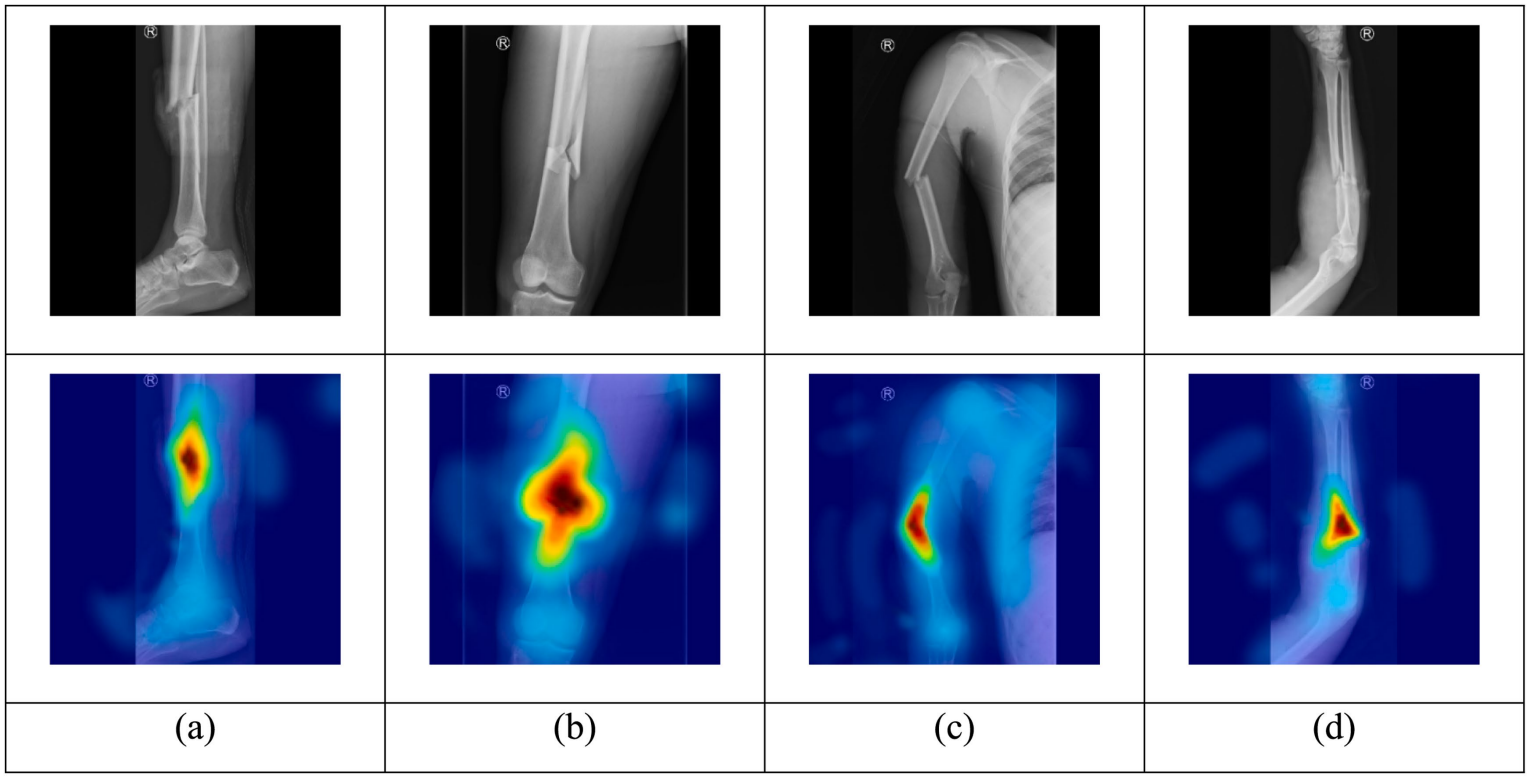
Model visualization using the Grad-CAM method for input images of **(a)** leg, **(b)** thigh, **(c)** upper arm, ad **(d)** lower arm.

Images of four classes of long bones were used to examine how the model makes predictions and which features it learns from input images. Results indicate that the trained model uses features such as edges, curvatures, discontinuities, and anomalies to align bone parts. These features help the model make predictions and reach a specific decision. Figure 7 shows the visualization of detected objects using the Grad-CAM method. The first row contains the original input images fed into the model, and the second row includes the Grad-CAM visualizations.

Figure 7a represents how the model used features from the input image of a leg and successfully identified the fractured area. Figure 7b indicates which part of the input image of a thigh the model used to extract features and make a prediction. Figure 7c is the visualization of the upper arm, and Figure 7d is the visualization of the lower arm. Taken together, these visualizations indicate the model uses relevant features from input images to make predictions. This clearly shows how the model makes predictions and uses similar features to make decisions like specialized medical practitioners.

**Figure 8:**
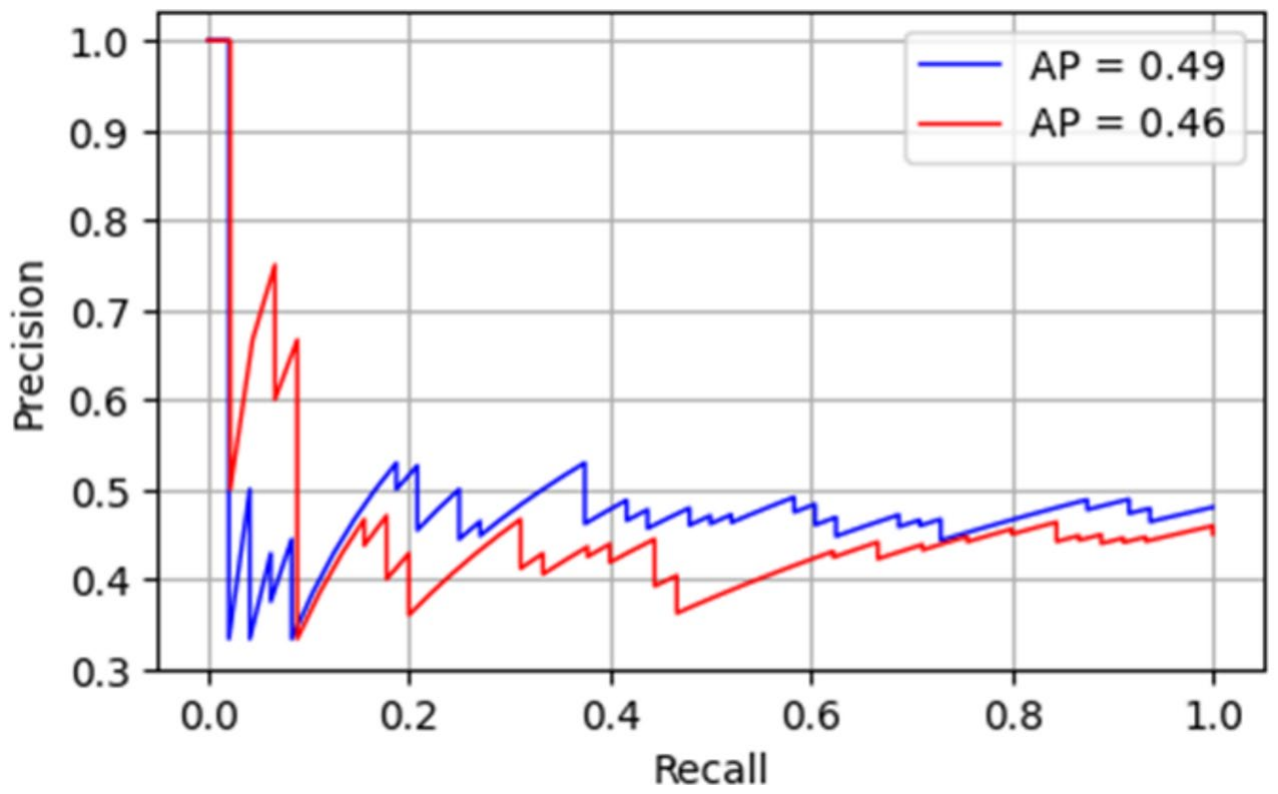
PR curve for standard R-CNN (blue) and adaptive (red) after randomization test.

In addition to Grad-CAM visualizations, quantitative sanity checks were conducted by randomizing labels and input images. The aim was to determine whether the model’s performance stemmed from learning true image-label associations rather than spurious correlations or memorization. Ground truth labels were randomly permuted, and images were replaced with noise-based proxies, and the model was retrained. The model trained on this corrupted dataset showed a near-chance performance. The resulting performance collapse confirms that the model does not rely on unintended shortcut features leaking into the labels. Figure 8 shows a PR curve of both standard Faster R-CNN and adaptive anchoring R-CNN recorded after randomization of labels and input images.

### 3.7 Fracture diagnosis with prescription recommendations

The rapid pace of progress has primarily influenced radiographic imaging in machine learning and deep learning. Over the last decade, the literature has witnessed an increasing number of studies applying deep learning to medical imaging. This application is crucial in enhancing the diagnosis process through medical imaging and mitigating the limitations of traditional approaches that rely solely on human interpretation. Developing countries, such as those in SSA, face enormous challenges in medical imaging. For example, Tanzania has 60 registered radiologists serving a population of approximately 60 million (Laage Gaupp et al., 2019). Although special programs have been established to train future radiologists (Iyawe et al., 2021). The shortage remains evident, particularly as the population continues to grow. Applying deep learning in medical imaging is essential and may serve as an additional intervention strategy.

There is a shortage of orthopedic surgeons in most developing countries in SSA, although limited evidence exists to quantify the exact gap. In Malawi, non-physician clinicians have been providing orthopedic care due to the shortage of orthopedists, and results indicate that task-shifting can be safe (Wilhelm et al., 2017). Deep learning models that assist in fracture diagnosis and provide prescription recommendations can significantly aid in resource-limited conditions, such as the SSA. Recommendation systems in the healthcare industry are gaining popularity as technology advances, aiming to enhance personalized healthcare. A guideline-driven decision support system to support family healthcare, utilizing semantic technology and open data analysis, has been introduced (Wang and Qian, 2021). An evidence-based clinical guideline system and monitored adherence to COVID-19 treatment recommendations have been implemented (Lichtner et al., 2023). An intelligent system that predicts a disease and recommends drugs by utilizing machine learning algorithms is proposed (Nayak et al., 2023). It is crucial to continue advancing deep learning models to reach their full potential, especially in medical applications such as diagnosis and treatment.

## 4 Conclusion

This study aimed to develop an efficient multi-class object detection model for bone fracture diagnosis that incorporates prescription recommendations. The research has also shown that the model’s efficiency and performance can be improved by modifying the anchoring process to search for areas where objects are likely to be located. Experiments have confirmed that applying adaptive anchoring in the process may reduce training time by up to 29%. This approach will help expand our understanding of how to continually improve object detection efficiency and performance. The study primarily confirmed that the object detection model can be utilized for bone fracture diagnosis and to suggest a corresponding prescription. Another important practical implication is that the study has identified the group most prone to bone fractures, the mechanisms of injury, and the locations of fractures, disaggregated by patient age. This provides valuable insight for law enforcement organizations in addressing the causes of bone fractures and for medical practitioners in treating them. The focus of this study was confined to long bones; notwithstanding this limitation, it offers valuable insights into the use of deep learning models as recommendation systems in medical applications.

## Data Availability

The original contributions presented in the study are included in the article/supplementary material, further inquiries can be directed to the corresponding author.
